# Val16A SOD2 Polymorphism Promotes Epithelial–Mesenchymal Transition Antagonized by Muscadine Grape Skin Extract in Prostate Cancer Cells

**DOI:** 10.3390/antiox10020213

**Published:** 2021-02-01

**Authors:** Janae D. Sweeney, Marija Debeljak, Stacy Riel, Ana Cecilia Millena, James R. Eshleman, Channing J. Paller, Valerie Odero-Marah

**Affiliations:** 1 Center for Cancer Research and Therapeutic Development and Department of Biological Sciences, Clark Atlanta University, Atlanta, GA 30314, USA; janae.sweeney@students.cau.edu (J.D.S.); amillena@cau.edu (A.C.M.); 2 Department of Pathology, The Sol Goldman Pancreatic Cancer Research Center, Johns Hopkins University School of Medicine, Baltimore, MD 21287, USA; mdebelj1@jhmi.edu (M.D.); Stacy_Riel@biocerna.com (S.R.); jeshlem@jhmi.edu (J.R.E.); 3 Department of Oncology, The Sol Goldman Pancreatic Cancer Research Center, Johns Hopkins University School of Medicine, Baltimore, MD 21287, USA; 4 The Sidney Kimmel Comprehensive Cancer Center, Johns Hopkins University School of Medicine, Baltimore, MD 21231, USA; cpaller1@jhmi.edu

**Keywords:** superoxide dismutase, epithelial–mesenchymal transition, prostate cancer, reactive oxygen

## Abstract

Epithelial–mesenchymal transition (EMT), a key event in cancer metastasis, allows polarized epithelial cells to assume mesenchymal morphologies, enhancing invasiveness and migration, and can be induced by reactive oxygen species (ROS). Val16A (Ala) SOD2 polymorphism has been associated with increased prostate cancer (PCa) risk. We hypothesized that SOD2 Ala single nucleotide polymorphism (SNP) may promote EMT. We analyzed SOD2 expression and genotype in various prostate cell lines. Stable overexpression of Ala-SOD2 or Val-SOD2 allele was performed in Lymph Node Carcinoma of the Prostate (LNCaP) cells followed by analysis of intracellular ROS and EMT marker protein expression. Treatments were performed with muscadine grape skin extract (MSKE) antioxidant, with or without addition of H_2_O_2_ to provide further oxidative stress. Furthermore, MTS cell proliferation, cell migration, and apoptosis assays were completed. The results showed that SOD2 expression did not correlate with tumor aggressiveness nor SOD2 genotype. We demonstrated that the Ala-SOD2 allele was associated with marked induction of EMT indicated by higher Snail and vimentin, lower E-cadherin, and increased cell migration, when compared to Val-SOD2 allele or Neo control cells. Ala-SOD2 SNP cells exhibited increased levels of total ROS and superoxide and were more sensitive to co-treatment with H_2_O_2_ and MSKE, which led to reduced cell growth and increased apoptosis. Additionally, MSKE inhibited Ala-SOD2 SNP-mediated EMT. Our data indicates that treatment with a combination of H_2_O_2_-generative drugs, such as certain chemotherapeutics and antioxidants such as MSKE that targets superoxide, hold promising therapeutic potential to halt PCa progression in the future.

## 1. Introduction

Accounting for nearly one in five new cancer-related diagnoses [1], prostate cancer (PCa) is the most common, non-cutaneous cancer and the second leading cause of cancer-related death among men nationwide [1]. Studies suggest that PCa mortality is due to distal metastasis [2] and tumor cell invasion [3]. Invasive metastatic cells are fundamentally characteristic of cancer cells that have undergone epithelial–mesenchymal transition (EMT) [4,5]. Specifically, EMT is defined as a developmental program that facilitates the conversion of cuboidal epithelial cells into motile mesenchymal phenotypes [6,7]. Furthermore, cadherin-switching is characteristic of EMT; in which the loss of adherens and tight junction proteins leads to the down regulation of E-cadherin and other epithelial markers, and the upregulation of key transcription factors and expression of mesenchymal markers such as N-cadherin [3], vimentin [7], and Snail [8]. Furthermore, in connection with EMT, Snail (SNAI1) is reportedly the transcription factor that leads to the migration and invasion of PCa cells [8].

Classically described as one of the hallmarks of cancer, oxidative stress is defined as an imbalance caused by low levels of protective antioxidant mechanisms and the accumulation of reactive oxygen species (ROS) [9,10]. ROS, primarily derived in the mitochondria [9], encompass free- and non-radicals that activate signal transduction pathways [11], both exacerbating oxidative stress and regulating multiple events such as cell cycle progression, migration, invasion [12], and apoptosis (in excessive amounts) [12]. As one of three isoforms of superoxide dismutase (SOD), SOD2 is known for its ability to act as both an oncogene [13] and tumor suppressor [14,15]. Representative of the gene, a non-synonymous single-nucleotide polymorphism (rs4880) is present at codon 16 of the SOD2 gene, encoding for either an alanine (Ala) or valine (Val) allele [16]. Literature reports that the SOD2 SNP affects the mitochondrial targeting signal (MTS) of SOD2 [17]. Specifically, studies suggest that the MTS of the Ala-SOD2 SNP forms an α-helical secondary structure, allowing SOD2 to be fully and more efficiently transported into the mitochondrial matrix; while the β-sheet structure [18] of the Val-SOD2 SNP causes SOD2 to be partially arrested within the translocase of the inner mitochondrial membrane (TIM 23 complex) [12,18,19]. Therefore, the Ala allele is 30–40% more active than the Val variant of the isoform, generating more active SOD2 and allowing for more efficient transport of SOD2 into the mitochondrial matrix [14,18,20]. Studies suggest that men with the SOD2 Ala/Ala genotype have increased susceptibility to aggressive PCa [21,22,23,24]. In addition, increased PCa risk has also been associated in men with the SOD2 Ala/Ala genotype who have low antioxidant status [14], high iron intake, or are smokers [23]; yet these men are known to often benefit more from compounds known to reduce oxidative stress, such as pomegranate and muscadine grape skin extract (MSKE) [25].

Epidemiologic studies suggest that diets rich in phytochemicals are associated with reduced risk of PCa [26]. MSKE is derived from the muscadine grape (*Vitis rotundifolia*) which is native to Southeastern United States and found anywhere from Delaware to the Gulf of Mexico and westward from Missouri to Texas [26]. The muscadine grape contains phytochemical properties distinct from other common red grapes used to produce red wine. The unique properties of MSKE include a predominance of anthocyanin 3,5-diglucosides, ellagic acid, and ellagic acid precursors [26]. Fruits rich in chemo-preventative agents such as anthocyanin have been shown to exhibit pro-apoptotic effects in several cell types in vitro. Moreover, it has been demonstrated that treatment with MSKE causes significant growth inhibition in well-established PCa cell lines (LNCaP); however, these inhibitory effects are not seen in normal primary prostate cells [26]. Furthermore, previous studies have shown that MSKE can inhibit PCa cell growth, induce apoptosis, and revert EMT by antagonizing superoxide [26,27].

Our investigation was driven by clinical trials conducted by Dr. Channing J. Paller and her team at Johns Hopkins University [28,29]. In a Phase II, placebo-controlled clinical trial, 112 patients with biochemically-recurrent PCa were randomly assigned to receive daily doses of eight capsules (4000 mg/day; high dosage) of Muscadine Plus (MPX), one capsule (500 mg/day; low dosage) of MPX plus seven capsules of placebo composed pulverized rice, or eight capsules of placebo [29]. Compared to the control arm, who experienced a 1.8-month median increase in prostate-specific antigen doubling time (PSADT), patients with the SOD2 *Ala/Ala* genotype experienced a 6.4-month median increase, demonstrating that *Ala/Ala* patients responded better to MSKE than those with *Val/Val* SNP [29].

In the present study, we hypothesized that the SOD2 *Ala/Ala* SNP is associated with enhanced EMT, potentially antagonized by MSKE in PCa. We focus on the establishment of SOD2 SNPs (Ala and Val) cell models in LNCaP PCa cells to delineate, in vitro, the mechanism(s) of action of the different allelic variants that may contribute to differential response to MSKE. We delineate that SOD2 *Ala/Ala* SNP but not SOD2 *Val/Val* SNP promotes EMT associated with increased cell migration. Although MSKE inhibits SOD2 *Ala/Ala* SNP-mediated EMT marker expression, it is not sufficient to inhibit proliferation, migration, or apoptosis, unless exogenous H_2_O_2_ is included.

## 2. Materials and Methods

### 2.1. Cell Culture, Reagents, and Antibodies

PCa cells used in this study and obtained from ATCC (Manassas, VA) included: RWPE-1 (normal transformed prostate epithelial tissue), LNCaP (derived from the left supraclavicular lymph node of Caucasian PCa patient), 22Rv1 (derived from a mice xenograft after propagation of castration-induced regression and relapse of parental CWR22), DU 145 (derived from brain metastasis of a Caucasian PCa patient), PC-3 (established in bone metastasis grade IV of Caucasian PCa patient), and MDA-PCa-2a and -2b (established from bone metastasis of an African American PCa patient). C4-2 (human bone fibroblast subline of LNCaP generated in immunocompromised mice), ARCaP-epithelial (ARCaP E; androgen-repressed cobblestone epithelial morphology cells derived from single cell cloning of parental ARCaP cells) and ARCaP-mesenchymal (ARCaP M; androgen-repressed spindle-shaped mesenchymal morphology derived from single cell cloning of parental ARCaP cells) were kind gifts from Dr. Leland Chung, Cedars-Sinai Medical Center, Los Angeles, CA, USA.

RWPE-1, LNCaP, 22Rv1, DU 145, PC-3, ARCaP E, and ARCaP M cells were grown in RPMI-1640 (Lonza, Alpharetta, GA). MDA-PCa-2a and -2b cells were grown in BRFF-HPC1 (Athena ES, Baltimore, MD, USA). All cells were supplemented with 10% or 20% (for MDA-PCa-2a/b) fetal bovine serum (FBS; Atlanta Biologicals, Inc., Flowery Branch, GA, USA) and 1% Penicillin/Streptomycin (Corning, Corning, NY, USA). Cells were maintained at 37 °C in a humidified incubator with 5% CO_2_. Geneticin (G418) was purchased from Calbiochem (Burlington, MA). The SOD2 gene cDNA (Val) ORF clone and mouse monoclonal anti-DYKDDDDK-tag antibody (FLAG) were obtained from Genscript (Accession No. NM _000636.3, Piscataway, NJ, USA). Nitrocellulose membrane was obtained from Bio-Rad (Hercules, CA). Roche cOmplete, EDTA-free protease inhibitor cocktail was from Sigma-Aldrich (Burlington, MA, USA). Anti-rabbit polyclonal SOD2 antibody, anti-mouse monoclonal β-Actin antibody, and donkey anti-goat secondary antibody were purchased from Santa Cruz Biotechnology (Dallas, TX, USA). Anti-rat monoclonal Snail antibody, and horseradish peroxidase (HRP)-linked goat anti-rat secondary antibody were from Cell Signaling Technology (Danvers, MA, USA). Goat monoclonal anti-vimentin antibody was from R&D Systems (Minneapolis, MN, USA). Mouse monoclonal anti-E-cadherin antibody was obtained from BD Biosciences (San Jose, CA, USA). MSKE was a kind gift from Dr. William Wagner, Muscadine Naturals (Clemmons, NC, USA). MSKE was reconstituted in 50% ethanol (EtOH) solution. HRP-conjugated sheep anti-mouse and donkey anti-rabbit secondary antibodies were purchased from GE Healthcare Life Sciences (Marlborough, MA, USA). Sterile dextran charcoal stripped fetal bovine serum (DCC) was obtained from GeneTex, Inc. (Irvine, CA, USA). MitoSOX Red Mitochondrial Superoxide Indicator was purchased from Invitrogen (Carlsbad, CA, USA).

### 2.2. Site-Directed Mutagenesis

Sample reactions (10, 50, and 100 ng) containing Val-SOD2 construct (Genscript, Piscataway, NJ) were utilized to conduct site-directed mutagenesis following manufacturer’s protocol (QuikChange Lightning Kit; Agilent Technologies, Santa Clara, CA, USA), using specific primers designed by Integrated DNA Technologies (IDT, Coralville, IA, USA) that would change Val-SOD2 cDNA (GTT; Val) construct to Ala-SOD2 cDNA (GCT; Ala). Success of mutagenesis was confirmed by DNA Sequencing at the Georgia State University DNA Sequencing Core (Atlanta, GA) using T7 Forward (5′- TAATACGACTCACTATAGGG-3′) and BGH Reverse (5′–TAGAAGGCACAGTCGAGG-3′) universal primers.

### 2.3. SOD2 SNP Stable Overexpression

LNCaP cells were stably transfected using Turbofect Transfection Reagent (Thermo Fisher Scientific, Waltham, MA, USA) according to manufacturer’s instructions. Briefly, 5 × 10^4^ LNCaP cells were plated in 24-well plates overnight. Subsequently, cells were transfected with 1 µg Neo (empty vector), Ala-SOD2, or Val-SOD2 cDNA. Selection for stable transfectants was performed in media containing 800 µg/mL G418, and thereafter 400 µg/mL G418 for maintenance. Selection of individual clones was performed using Bel-Art Cloning Discs (Fisher Scientific, Pittsburgh, PA, USA). Success of transfections was confirmed through probing with the DKYDDDDK FLAG-tag antibody by western blot analysis. Of note, all the experiments were performed with the 3 clones for Al/Ala and the 3 clones for Val/Val, but results were shown for representative Ala/Ala clone A3 and Val/Val clone D2.

### 2.4. ROS Assay

SOD2 SNP-overexpressing LNCaP cells (1 × 10^4^) were plated in black 96-well plates and incubated with ROS indicator (2′, 7′-dichlorofluorescin diacetate- DCFDA dye) and/or Total ROS/Superoxide Detection Solution (Enzo Life Sciences, Farmingdale, NY, USA) for 1 h at 37 °C and 5% CO_2_. Following incubation, intracellular levels of H2O2 was detected using the BioTek Synergy H1 Hybrid Reader at 485 excitation/535 emission. Fluorescence of Total ROS and Superoxide was detected using standard fluorescein (488 excitation/520 emission) and rhodamine (550 excitation/610 emission) filter sets, respectively.

### 2.5. Immunofluorescence

LNCaP cells (5 × 10^3^) overexpressing SOD2 SNPs were plated in a 8-well chamber slide (Thermo Fisher Scientific, Waltham, MA, USA) overnight before being fixed with 1:1 methanol:ethanol solution. Following fixation, cells were washed with 1X Phosphate Buffer Saline (PBS) and blocked with 5% goat serum. Subsequently, slides were incubated for 1 h with primary FLAG antibody (DYKDDDDK tag) in antibody diluent solution (Dako, Carpinteria, CA, USA). Slides were then incubated with secondary anti-mouse antibody (Alexa Fluor 488) for 1 h in the dark at room temperature, before incubating with MitoSOX to visualize the production of superoxide in the mitochondria for 10 min. Slides were then counterstained with DAPI or 4′,6-diamidino-2-phenylindole (1 µg/mL; Santa Cruz Biotechnology, Dallas, TX, USA). Slides were mounted using Fluoro-Gel mounting medium (Electron Microscopy Sciences, Hatfield, PA, USA) and fluorescence microscopy performed using Zeiss microscope and ZEN Imaging Software.

### 2.6. Treatments

The 4–5 × 10^5^ cells were plated in a 6-well cell culture plate overnight. The next day, cells were serum-starved for 3 h in phenol/serum-free RPM- 1640 (Corning, Corning, NY, USA), and then grown under experimental conditions in phenol/serum-free RPMI 1640 containing 5% dextran charcoal stripped fetal bovine serum (DCC) for 0–72 h. Experimental conditions included untreated, ethanol control (EtOH; 0.005%), H_2_O_2_ (250 µM), MSKE (20 µg/mL), and 250 µM H_2_O_2_ + 20 µg/mL MSKE. Subsequently, we collected protein lysate for western blot analysis, or performed cell proliferation/migration/apoptosis assays.

### 2.7. Annexin V/Dead Cell Apoptosis Assay

Cells were evaluated for programmed cell death using Alexa Fluor 488 Annexin V/Dead Cell Apoptosis kit (Invitrogen, Carlsbad, CA, USA). Apoptosis was assessed by double staining cells with Alexa Fluor 488 Annexin V/Propidium iodide (PI) and performing flow cytometry utilizing the Accuri C6 Flow Cytometer (Accuri Cytometers, Inc., Ann Arbor, MI, USA). Cells that stained negative for both Alexa Fluor 488 Annexin V and PI (Annexin V−/PI−) were considered non-apoptotic/viable; cells that stained positive for Alexa Fluor 488 Annexin V and negative for PI (Annexin V+/PI−) were considered to undergoing early apoptosis. Cells that stained positive for both Alexa Fluor 488 Annexin V and PI (Annexin V+/PI+) were considered as undergoing late apoptosis; cells that stained negative for Alexa Fluor 488 Annexin V and positive for PI (Annexin V−/PI+) were considered to be necrotic. Apoptosis experiments were performed in triplicate, and representative results displayed in the form of graphs.

### 2.8. Cell Viability Assay

Cells were seeded at a density of 2 × 10^3^ cells/well in 96-well plates and allowed to attach overnight. Cell viability was assessed according to the manufacturer’s protocol using the CellTiter 96^®^ Aqueous One Solution Cell Proliferation Assay (Promega, Madison, WI). Viability was measured at Day 0, and 3 using the BioTek Synergy H1 Hybrid Reader at 490 nm absorbance.

### 2.9. Cell Migration Assay

Cell migration was analyzed utilizing Costar 24-well plates (Corning, Corning, NY, USA) containing polycarbonate filter inserts with an 8 µm pore diameter size (Fisher Scientific, Pittsburgh, PA, USA). These wells were coated with 4.46 µg/µL rat tail collagen I (BD Biosciences, San Jose, CA, USA) on the outside and incubated for 24 h at 4 °C. 5 × 10^4^ cells were plated in the upper chamber containing phenol/serum-free RPMI 1640 containing 5% dextran charcoal stripped fetal bovine serum (DCC); whereas the bottom chamber contained phenol/serum-free RPMI 1640 supplemented with 5% dextran charcoal stripped fetal bovine serum (DCC) and various experimental conditions which included: untreated, ethanol control (EtOH; 0.005%), H_2_O_2_ (250 µM), MSKE (20 µg/mL), and 250 µM H_2_O_2_ + 20 µg/mL MSKE. After 48 h, cells that migrated to the bottom of the insert were fixed with 10% Formalin (Formaldehyde and 1X PBS), stained with 0.05% Crystal Violet, and counted to obtain the relative cell migration.

### 2.10. Western Blot Analysis

Whole protein cell lysates were collected in Lysis buffer (1X modified RIPA buffer, 1X Protease inhibitor, 1 mM phenylmethylsulfonyl fluoride (PMSF), and 1mM sodium orthovanadate). For each protein sample, 30 μg protein was run on a 10% SDS-PAGE gel and transferred to 0.45 μm nitrocellulose membrane (Bio-Rad, Hercules, CA, USA). The nitrocellulose membranes were then blocked in 5% Milk (Quality Biological, Inc, Gaithersburg, MD, USA) with TBS (1M Tris, 5 M NaCl, 0.05% BSA) or 5% Milk with TBST (TBS + 0.5% Tween-20); then subsequently incubated with appropriate primary antibodies overnight in 4 °C. Membranes were incubated at room temperature for 2 h with the appropriate secondary antibody followed by visualization using Luminata Forte Western HRP Substrate (Millipore-Sigma, Burlington, MA) and visualized by Bio-Rad ChemiDoc Imaging System to detect protein expression. The membranes were stripped using mild stripping buffer (0.75% Glycine, 0.05% SDS, 0.5% Tween-20 at pH 2.2) prior to re-probing with a different antibody.

### 2.11. SOD2 Genotyping by Pyrosequencing

Pyrosequencing of the SOD2 gene was performed by PCR amplifying the gene using 100 ng genomic DNA and 5′-TGTAAAACGACGGCCAGTACTGACCGGGCTGTGCTT-3′ (SOD2 Forward M13) and 5′-biotin~CAGGAAACAGCTATGACCGCGTTGATGTGAGGTTCCAG-3′ (SOD2 Reverse M13 Biotin) at 10 µM in Platinum PCR SuperMix HiFi (Thermo Fisher Scientific, Waltham, MA) and thermocycled for 1 min at 96 °C, 30 s at 95 °C, and 30 s at 62 °C for 35 cycles. The bottom strand was captured and pyrosequenced using the SOD2 Sequencing primer (5′-AGCAGGCAGCTGGCTCCG-3′) in a PyroMark Q24 Instrument (Qiagen, Germantown, MD, USA) according to the manufacturer’s instructions. Sequencing traces were interpreted at the Johns Hopkins Molecular Diagnostics Laboratory as homozygous wild-type, heterozygous, or homozygous variants.

### 2.12. Quantitative Real-Time RT-PCR Analysis

Total RNA was isolated (Qiagen, Germantown, MD, USA), and reverse transcribed using Molony Murine Leukemia Virus Reverse Transcriptase (M-MLV RT) and oligo dT primers (Invitrogen, Carlsbad, CA, USA). Resulting cDNA samples were amplified by quantitative real time RT-PCR (Bio-Rad CFX Connect) in the presence of SYBR Green PCR Master Mix (Applied Biosystems, Beverly, MA) using qPCR primers (IDT, Coralville, IA, USA) specific for SOD2 (Forward 5′-GCCTACGTGAACAACCTGAA-3′; Reverse 5′-GCCGTCAGCTTCTCCTTAAA-3′ All data were normalized to glyceraldehyde 3-phosphate dehydrogenase (GAPDH; Forward 5′-GAAGGTGAAGGTCGGAGTC-3′; Reverse 5′-GAAGATGGTGATGGGATTTC-3′).

### 2.13. Statistical Analysis

All experiments in the present study were performed at least in triplicate. Statistical analysis of data and graphs were generated using GraphPad Prism 8 Software (San Diego, CA, USA). Values of statistical significance were described through the use of asterisks to denote *p*-value; not significant: *p* > 0.05 (ns), significant: *p* < 0.05 (*), very significant: *p* < 0.01 (**), extremely significant: *p* < 0.001 (***), *p* < 0.0001 (****).

## 3. Results

### 3.1. SOD2 Protein Expression and Genotype in Prostate Cancer Cell Lines

**Figure 1:**
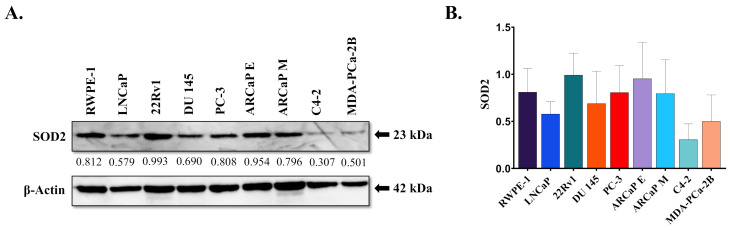
Overview of Superoxide Dismutase 2 (SOD2) protein expression in prostate cancer cell (PCa) panel. (**A**) SOD2 expression was probed using western blot analysis of whole cell lysate (30 µg/mL) in a prostate cell panel. β-Actin was used as a loading control. (**B**) Quantification of western blot analysis was performed using Image J (National Institutes of Health- NIH) normalized to β-Actin. Results are representative of triplicate experiments performed at least twice independently.

To study the expression of SOD2 in prostate cells, we performed western blot analysis, using known normal transformed prostate epithelial (RWPE-1) and PCa (LNCaP, 22Rv1, DU 145, PC-3, ARCaP E, ARCaP M, C4-2, and MDA-PCa-2b) cell lines. Western blot analysis revealed that SOD2 was present in all probed cell lines (Figure 1A,B). However, although subtle differences were observed in protein expression, there was no statistical significance in the different prostate cancer cell lines (Figure 1A,B). Therefore, expression of total protein does not appear to correlate with degree of tumor aggressiveness.

To further investigate SOD2, we isolated genomic DNA from these cell lines for next-generation SNP genotyping analysis. Pyrosequencing revealed that most PCa cell lines were heterozygous (*Ala/Val*) for SOD2 SNPs (Table 1). However, several of the heterozygous cell lines had extra copies of alleles, suggesting multiple copy numbers. Of the PCa cell lines analyzed, only metastatic MDA-PCa-2a and -2b contained homozygous (*Ala/Ala*) SOD2 SNPs. Together, this data shows that overall protein expression and genotype of the SOD2 *Ala/Ala* and *Val/Val* SNPs are variable depending on the prostate normal or cancerous cell type.

### 3.2. Ala-SOD2 SNP Promotes EMT

**Figure 2:**
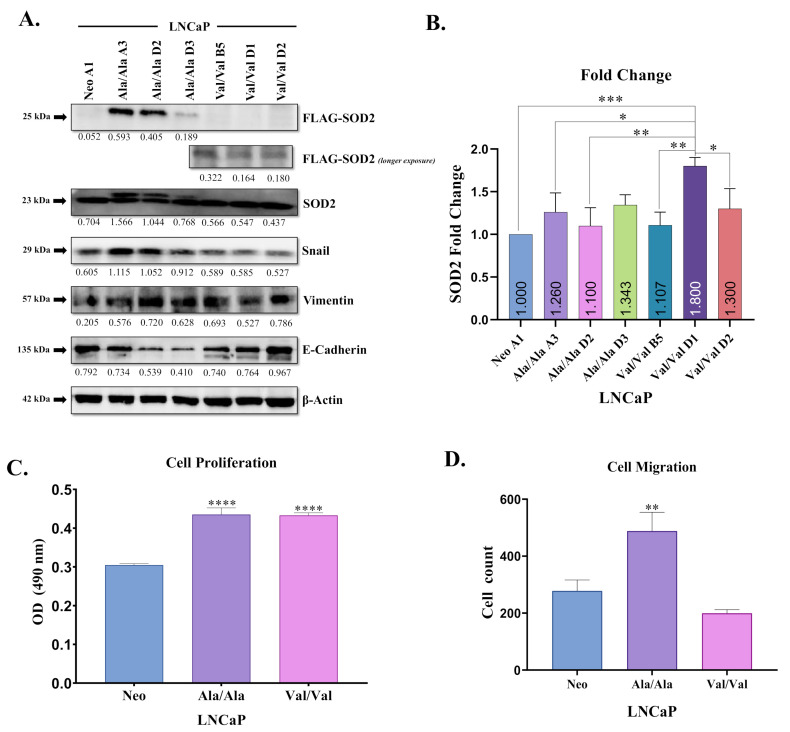
Overexpression of Ala-SOD2 SNP complementary DNA (cDNA) in LNCaP cells leads to increased epithelial-mesenchymal transition (EMT), cell growth, and migration. Ala-, Val-SOD2, or empty vector (Neo) cDNA, were stably transfected into LNCaP cells. (**A**) Western blot analysis of FLAG-SOD2, total SOD2 (exogenous and exogenous) and EMT markers (Snail, vimentin, E-cadherin) was performed. Data indicated that Ala-SOD2 (*Ala/Ala*)-expressing cells are more effective than Val-SOD2 (*Val/Val*)-expressing cells, at inducing EMT (higher Snail and vimentin; lower E-cadherin). β-Actin was used as a loading control. (**B**) Real-time polymerase chain reaction (PCR) analysis was performed for stable transfectants of LNCaP cells with Neo, Ala- or Val-SOD2 cDNA (*Ala/Ala* and *Val/Val*). Baseline (**C**) cell proliferation and (**D**) cell migration in untreated Neo (empty vector) control and SOD2-overexpressed LNCaP cells were analyzed. Statistical significance (one-way analysis of variation -ANOVA) of differences between Neo control and SOD2 SNP-overexpressed cells was determined using GraphPad Prism *p* < 0.05 (*), *p* < 0.01 (**), *p* < 0.001 (***), *p* < 0.0001 (****). Results are representative of triplicate experiments performed at least twice independently.

To further study SOD2 SNPs in PCa cell lines, we first performed transient overexpression in PCa cells. LNCaP cells were transiently transfected with Neomycin (empty vector Neo), Ala-SOD2, or Val-SOD2 FLAG-tagged cDNA generated through site-directed mutagenesis. Previously in our lab, we examined the expression of EMT markers in a PCa cell panel [30]. It was shown that the normal transformed prostate epithelial cell line (RWPE-1) and tumorigenic but non-metastatic Caucasian American (CA) PCa cell lines, LNCaP and 22Rv1, expressed low levels of Snail and vimentin (mesenchymal markers) and high levels of E-cadherin (epithelial marker) [30]. In contrast, metastatic CA PCa cell lines DU 145, PC-3, ARCaP E, and ARCaP M expressed high levels of Snail and vimentin and low levels of E-cadherin. Interestingly showing the same trend, tumorigenic but non-metastatic African American (AA) cell line, MDA-PCa-2b, also showed high levels of Snail and vimentin, but also high levels of E-cadherin [30]. In the present study, western blot analysis of transiently transfected SOD2-SNP cells showed successful overexpression of Ala- and Val-SOD2 SNPs at 48 and 72 h; however, EMT marker expression did not show differences (Supplementary Figure S1A). Therefore, we performed stable transfection with Neo, Ala-SOD2, or Val-SOD2 FLAG-tagged cDNA. Representative clones were isolated with cloning rings, and each was assigned an arbitrary letter code *A*–*D* and number 1–5 to denote different clones. Western blot analysis examining protein expression of FLAG-SOD2 (DYKDDDDK FLAG-tag) in LNCaP cells showed minimal exogenous SOD2 expression in the Neo clone, higher expression in Ala-SOD2 clones, and lower expression in Val-SOD2 clones which was more visible with longer exposure (Figure 2A, Supplementary Figure S2A). This agrees with prior literature that Ala-SOD2 is more stable than Val-SOD2, as it is efficiently transported into the inner mitochondrial matrix membrane [14]. Further analysis of our western blot analysis also showed that increased induction of EMT (higher Snail and vimentin, and lower E-cadherin) was observed in Ala-SOD2 SNP clones when compared to Neo and Val-SOD2 clones (Figure 2A, Supplementary Figure S2B). Next, RT-PCR analysis of representative clones showed that although there was a trend towards increased SOD2 mRNA expression in all SOD2-overexpressed clones, there was only statistically significant increases seen in Val -SOD2 clones when compared to the Neo (empty vector) control (Figure 2B), suggesting that differences observed in protein may be post-transcriptional. To investigate the biological role of SOD2 SNPs, we performed a baseline cell viability assay using the CellTiter 96^®^ AQueous One Solution Cell Proliferation MTS Assay. MTS analysis of representative clones (Neo control, Ala-SOD2, and Val-SOD2) demonstrated that over a three-day period, overexpression of Ala- and Val-SOD2 SNPs in LNCaP cells led to significantly increased cell viability (*p* < 0.0001) compared to the Neo control (Figure 2C). In addition to the cell viability assay, we performed a cell migration utilizing the Boyden chamber, or Transwell method. Baseline analysis of cell migration in our representative clones (Neo control, Ala-SOD2, and Val-SOD2) demonstrated that, over a 48 h period, SOD2 overexpression led to a significant migratory increase in Ala-SOD2 cells (*p* < 0.05) when compared to the Neo control and Val-SOD2 clone (Figure 2D). Overall, these results suggest that overexpression of Ala-SOD2 SNP may play a role in the induction of EMT, associated with increased cell proliferation and migration. However, both SOD2 SNPs are associated with increased cell viability but not cell migration in LNCaP cells.

### 3.3. Overexpression of Ala-SOD2 SNP Leads to Increased Levels of Total ROS and Superoxide Compared to Val-SOD2 SNP

**Figure 3:**
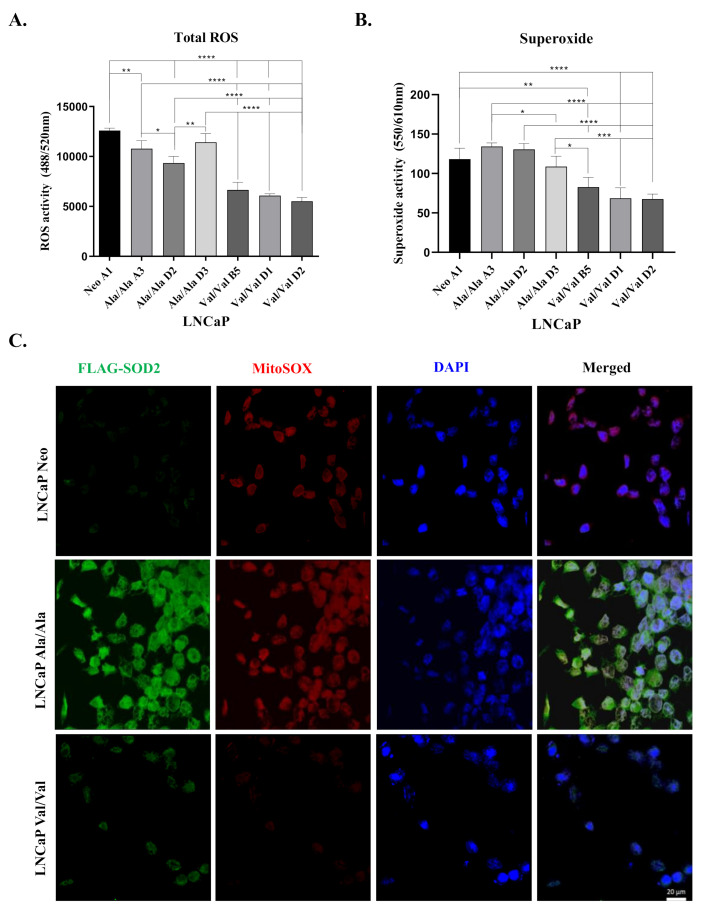
Stable overexpression of Ala-SOD2 SNP displays higher reactive oxygen species (ROS) compared to Val-SOD2 SNP in prostate cancer cells. (**A**) Total ROS levels, which includes overall oxidative stress in the form of intracellular levels of hydrogen peroxide, hydrogen peroxide (H_2_O_2_), peroxynitrite (ONOO^−^), hydroxyl radicals (HO), nitric oxide (NO), and peroxy radical (ROO), were measured using the ROS-ID Total ROS/Superoxide Detection kit in LNCaP cells that were stably transfected with Ala- or Val-SOD2 cDNA. (**B**) Superoxide (O_2_^−^) levels were measured using Superoxide Detection Solution from the ROS-ID Total ROS/Superoxide Detection kit. (**C**) LNCaP Neo and SOD2 SNP-overexpressing cells were utilized for immunofluorescent analysis of exogenous SOD2 using FLAG antibody (green), superoxide using MitoSOX stain (red), and 4′,6-diamidino-2-phenylindole (DAPI) (blue) for nucleus. Cells overexpressing Ala-SOD2 (*Ala/Ala*) were shown to have higher levels of FLAG and superoxide when compared to Neo and Val-SOD2 (*Val/Val)* cells. The statistical significance of differences (one-way ANOVA) for the ROS and superoxide data was analyzed by GraphPad Prism and shows *p* < 0.05 (*), *p* < 0.01 (**), *p* < 0.001 (***), *p* < 0.0001 (****) from experiments performed in triplicate at least twice independently. Magnification 40×. Scale bar = 20 µm.

To investigate basal levels of intracellular ROS in LNCaP cells that were stably transfected with SOD2 Ala and Val SNPs, we performed ROS assays. Overall oxidative stress was assessed in experimental cells via analysis of intracellular levels of total ROS (hydrogen peroxide: H_2_O_2_, peroxynitrite: ONOO^−^, hydroxyl radicals: HO, nitric oxide: NO, and peroxy radical: ROO and superoxide: O_2_^−^) or superoxide. Stable transfection with the Ala-SOD2 SNP led to significantly higher total ROS (Figure 3A) and higher superoxide when compared to Val-SOD2 SNP clones (Figure 3B). However, we observed that there were lower levels of total ROS and superoxide (not significantly) in both Ala- and Val-SOD2 clones when compared to the Neo control that may be attributed to endogenous SOD2 (Figure 3A,B). Immunofluorescent visualization of exogenous SOD2 (utilizing the FLAG antibody; green), mitochondrial superoxide (utilizing MitoSOX stain; red), and nuclei (DAPI) was performed in representative clones which illustrated that cells overexpressing the Ala-SOD2 SNP exhibited higher levels of FLAG and superoxide compared to Neo and Val-SOD2 clones, suggesting that Ala-SOD2 increases levels of superoxide (Figure 3C, Supplementary Figure S3A,B). Supplementary data of the transient overexpression of SOD2 SNPs further supported co-localization of the MitoSOX stain (red) and SOD2 antibody (green) in the mitochondria of SOD2 SNP-overexpressed cells. Specifically, Ala-SOD2 showed greater co-localization when compared to Val-SOD2 (Supplementary Figure S1B). These results imply that Ala-SOD2 SNP leads to increased total ROS and superoxide when compared to the Val-SOD2 SNP. Surprisingly, this is a clear departure from studies showing that Ala-SOD2 having higher dismutase activity compared to Val-SOD2.

### 3.4. MSKE Inhibits SOD2 SNP-Mediated EMT

**Figure 4:**
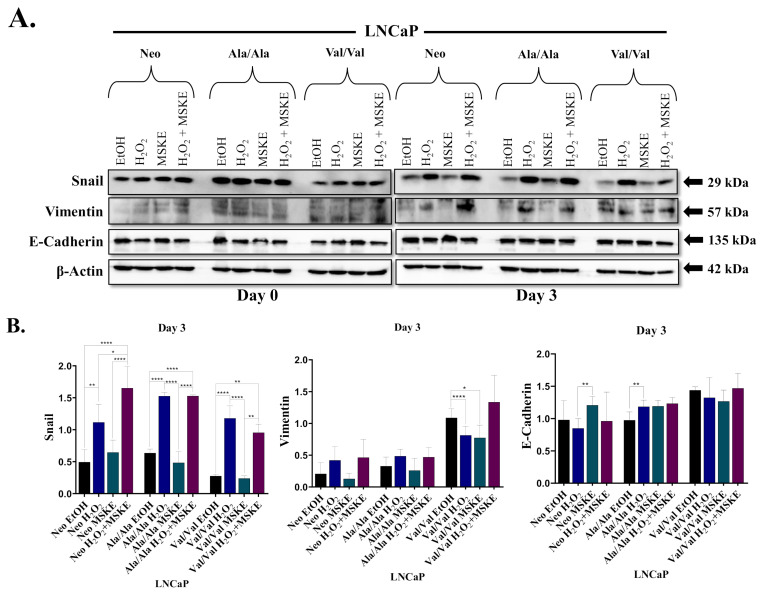
EMT can be inhibited in LNCaP cells overexpressed with SOD2 SNPs. LNCaP Neo, *Ala/Ala*, or *Val/Val* were treated with 5% dextran charcoal stripped fetal bovine serum (DCC) containing ethanol (EtOH) control, 250 µM hydrogen peroxide (H_2_O_2_), 20 µg/mL muscadine grape skin extract (MSKE), or H_2_O_2_ + MSKE combination of the same concentration from day zero to day three. Subsequently, (**A**) western blot analysis of whole cell lysate was performed using EMT markers Snail, vimentin and E-cadherin. β-Actin was used as a loading control. (**B**) Quantification of western blots was performed using Image J (National Institutes of Health-NIH) normalized to β-Actin. The statistical significance of differences (one-way ANOVA and paired t-test) for Image J data was analyzed by GraphPad Prism and shows *p* < 0.05 (*), *p* < 0.01 (**), *p* < 0.0001 (****). Results are representative of triplicate experiments performed at least twice independently.

Previously in our lab, we demonstrated that MSKE was able to revert Snail-mediated EMT in PCa cells by antagonizing superoxide species [31]. Since superoxide is increased in cells overexpressing Ala-SOD2, we investigated whether MSKE would antagonize Ala-SOD2 SNP-mediated EMT. In the present study, we showed (by western blot analysis) that by day three of treatments, the Ala-SOD2 clone showed decreases in both Snail and vimentin expression after treatments with MSKE, when compared to the Ala-SOD2 Day 0 EtOH control and MSKE treatment (Figure 4A,B, Supplementary Table S1). This trend of decreased Snail expression was also exhibited in Val-SOD2 SNP cells when compared to the Val-SOD Day 0 EtOH and MSKE treatment (Figure 4A,B, Supplementary Table S1). E-cadherin expression was shown to increase, following MSKE treatment, for the Neo control and SOD2-SNP representative clones when compared to the Day 0 EtOH and MSKE treatment of each clone type. These results show that MSKE can inhibit EMT in Ala-SOD2 SNP-overexpressing LNCaP cells.

### 3.5. Co-Treatment with H_2_O_2_ and MSKE Decreases Cell Proliferation and Increases Apoptotic Activity

**Figure 5:**
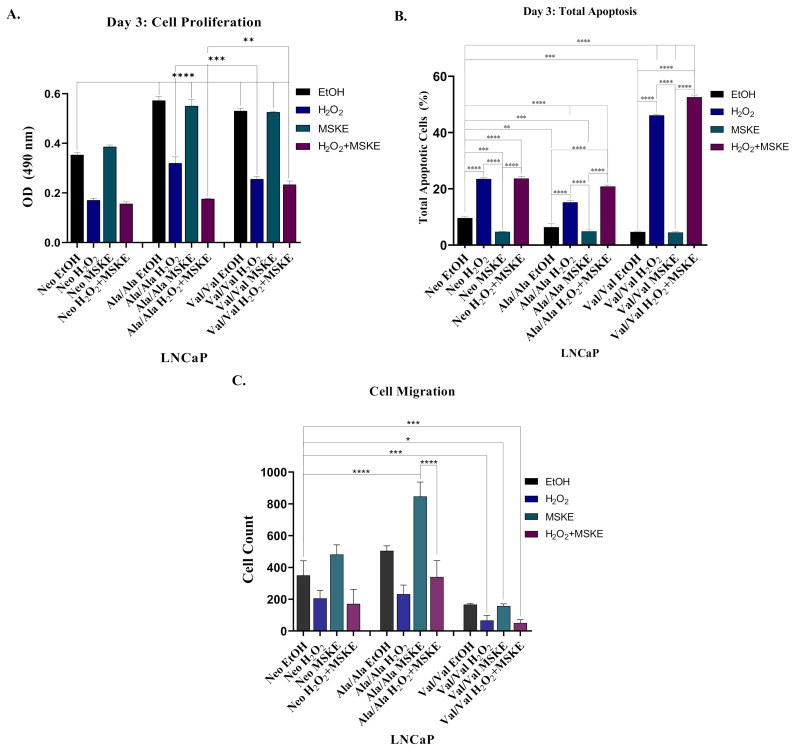
Rate of cell viability, apoptosis and migration is affected by H_2_O_2_ + MSKE in LNCaP cells overexpressing SOD2 SNPs. (**A**) 3-(4,5-dimethylthiazol-2-yl)-5-(3-carboxymethoxyphenyl)-2-(4-sulfophenyl)-2H-tetrazolium) (MTS) cell proliferation in SOD2 SNP-overexpressed LNCaP cells was measured following treatment with H_2_O_2_ and/or MSKE. The combination treatment of H_2_O_2_ and MSKE was shown to decrease cell viability. (**B**) Total apoptosis measured in cells treated indicated that at day three, the combination treatment of H_2_O_2_ and MSKE induced apoptosis. (**C**) Cell migration was determined across transwells coated with type I rat-tail collagen. The significance of differences (one-way ANOVA) between Neo control cells treated with EtOH, and cells treated with H_2_O_2_ + MSKE was analyzed using GraphPad Prism and shows *p* < 0.05 (*), *p* < 0.01 (**), *p* < 0.001 (***), *p* < 0.0001 (****). Results are representative of experiments performed in triplicate at least twice independently.

We have previously described that MSKE promotes apoptosis in C4-2 PCa cells [27]. H_2_O_2_ has also been shown to decrease cell proliferation and send cells towards apoptosis [32]. We sought to examine whether MSKE would antagonize Ala-SOD2 and/or Val-SOD2 SNP-mediated cell proliferation/migration and affect apoptosis. Representative SOD2-SNP stably transfected LNCaP cells were treated with both individual and coupled treatments of H_2_O_2_ and MSKE; with EtOH serving as an experimental control. An MTS assay performed over a three-day period showed that treatment with H_2_O_2_ alone and H_2_O_2_ coupled with MSKE led to significant decrease in cell proliferation for Neo, Ala-SOD2 and Val-SOD2 representative clones, when compared to EtOH controls (*p* < 0.0001) (Figure 5A, Supplementary Figure S4). However, MSKE alone did not affect cell viability (Figure 5A, Supplementary Figure S4). However, the coupling of H_2_O_2_ and MSKE showed a greater decrease in cell viability compared to H_2_O_2_ alone, for only Ala-SOD2 SNP-overexpressed cells, suggesting an additive effect (Figure 5A, Supplementary Figure S4). Next, apoptotic analysis of treated cells was performed. The use of 20 µg/mL MSKE has been previously cited in literature to promote apoptosis in PCa cells [26]. Interestingly, we observed that by day three of treatment, coupling 20 µg/mL MSKE with H_2_O_2_ (H_2_O_2_ + MSKE) induced significantly increased total apoptosis in Ala-SOD2 and Val-SOD2 cells at *p* < 0.0001, when compared to Neo-, Ala-SOD2-, and Val-SOD2 EtOH-treated cells, respectively (Figure 5B). Therefore, co-treatment of H_2_O_2_ and MSKE in SOD2 SNP-overexpressed LNCaP cells promotes apoptosis, while also decreasing cell proliferation. In our analysis of cell migration, when compared to the EtOH control, significant decreases were seen in Ala-SOD2 and Val-SOD2 cells treated with H_2_O_2_ alone, that were not decreased any further by combination H_2_O_2_ + MSKE (Figure 5C). To our surprise, MSKE alone did not decrease cell migration and in fact, Ala-SOD2 cells showed a significant increase in migration (Figure 5C, Supplementary Figure S5). Altogether, this data suggests that SOD2-overexpressed cells may benefit more from treatment with H_2_O_2_ alone or in combination with MSKE, rather than MSKE alone as originally hypothesized.

## 4. Discussion

Superoxide dismutase 2 (SOD2) polymorphisms have been implicated in several diseases including diabetes mellitus [33,34,35], Alzheimer’s disease [36], heart disease [35], and cancers; including breast [37] and PCa [21]. As evidenced in previous studies, individuals with the Ala variant of the SOD2 SNP are vulnerable to increased risk of aggressive PCa when compared to non-Ala carriers [21]. Susceptibility increases in these patients when they have low lycopene status [14] or are chronic cigarette smokers [23]. This characteristic of the Ala variant may be attributed to reports that the Ala variant is 30–40% more active than the Val variant, allowing for increased amount of SOD2 to be shuttled into the inner mitochondrial matrix when associated with the Ala SNP [14,18,20]. The goal of this study was to create an LNCaP SOD2 SNP model (*Ala/Ala* and *Val/Val*) in order to determine the in vitro mechanism of action of the SOD2 SNPs, and further explain variance in patient response to antioxidant treatment.

Preliminarily, we explored the expression of SOD2 in prostate cell lines. Our lab observed subtle but no significant differences in SOD2 protein expression in the various PCa cell lines (Figure 1). Based on previous findings of EMT in these cell lines [30], it appears that the levels of SOD2 protein does not correspond with the levels of EMT markers. For example, LNCaP and MDA-PCa-2b, which are on different spectrums of aggressiveness, expressed similar levels of SOD2, yet LNCaP cells display low EMT and MDA-PCa-2b expressed more EMT (high Snail and vimentin) [30]. Additionally, although SOD2 protein expression is relatively similar between the two cell lines, they exhibited different alleles after pyrosequencing. Therefore, at this time we are unable to determine a correlation between SOD2 protein expression in PCa cell lines and the SNP genotype expressed, suggesting more complex interactions at play.

Our investigation of SOD2 genotypes, via pyrosequencing, allowed us to demonstrate allelic variation amongst prostate cell lines. Of the PCa cell lines analyzed, we observed that only metastatic MDA-PCa-2a and -2b contained homozygous WT (*Ala/Ala*) SOD2 SNPs (Table 1). Most PCa cell lines were heterozygous (*Ala/Val*) for SOD2 SNPs. However, several of the heterozygous cell lines had extra copies of alleles, suggesting multiple copy numbers (Table 1). The extra copies of alleles of SOD2 SNPs on chromosome 6q25.3 may be attributed to chromosomal aberrations in PCa cell lines. Spectral karyotyping (SKY) revealed that tetraploid LNCaP cells [38,39] carried translocated chromosomes from three reciprocal translocation events—t(1;15), t(4;6) and t(6;16) [38]. It is possible that the two translocation events—t(4;6) and t(6;16)—could affect allelic variation, causing extra alleles to be observed on chromosome 6 where the SOD2 gene is located. Other chromosomal translocations were reported in PC-3 and DU 145 PCa cell lines. Both of these cell lines were reported to be aneuploid, with extra chromosomes at several positions within their respective karyotypes [39]. Although aneuploid, SKY analysis in PC-3 cells did not reveal any chromosomal aberrations on chromosome 6 [39]. This may explain why SOD2 SNP pyrosequencing showed just two alleles (*Val/Val*) for PC-3 cells. Interestingly, the only PCa cell lines that were homozygous *Ala/Ala* for SOD2 were MDA-PCa-2a and -2b. These cell lines can switch from diploid to aneuploidy with high passage numbers [40]; however, we utilized low passage numbers for this study, which could explain why no extra alleles were seen in pyrosequencing results (Table 1).

Previous studies have reported the capability of SOD2 to induce EMT via the production of H_2_O_2_ in pancreatic cancer cells [41]. We sought to understand whether induction of EMT would vary among the Ala and Val variants of SOD2. Here, we demonstrated that, in comparison to Neo control, both Ala- and Val-SOD2 SNPs increased cell proliferation when overexpressed in the LNCaP PCa cell line. However, the Ala-SOD2 was more effective at promoting EMT characterized by high Snail and vimentin and low E-cadherin protein levels, associated with increased cell migration (Figure 2A,C,D). Our RT-PCR analysis of SOD2 SNP-overexpressed LNCaP cells yielded results that demonstrated an increase in SOD2 mRNA expression in all SOD2-overexpressed clones. Despite this, statistically significant increases were only observed in seen in Val -SOD2 clones when compared to the Neo (empty vector) control (Figure 2B). We found this interesting, as we anticipated Val-SOD2 SNP cells would experience mRNA instability, since studies have reported that import of Val protein associated with SOD2 is partially arrested in the inner mitochondrial matrix and subject to proteasomal degradation [18,20]. However, in line with our findings in the present study, another study focusing on SOD2 SNP-overexpression in mouse embryo fibroblasts (MEFs), also demonstrated that Val-SOD2 MEFs expressed increased mRNA levels when compared to Ala-SOD2 overexpressed cells lines; however, Ala-SOD2 MEFs displayed higher SOD2 protein levels per mRNA ratio suggesting an imbalance in protein production [42]. Surprisingly, intracellular ROS analysis indicated increased total ROS and superoxide in Ala-SOD2-overexpressed cells when compared to Val-SOD2 SNP-overexpressed cells. Total ROS was still lower than Neo control, suggesting there is some increased dismutase activity when these SNPs are overexpressed. However, we expected Ala-SOD2 to display more dismutase activity based on reports that Ala-SOD2 variant is more active than the Val-SOD2 variant, due to efficient transport of SOD2 into the inner mitochondrial matrix [14,18,20]. This was further supported by immunofluorescence that also demonstrated a large disparity of superoxide (as shown by staining with MitoSOX; red) between Ala and Val SNP-overexpressed cells, with Ala-SOD2 overexpressing cells displaying higher superoxide compared to Neo and Val-SOD2 cells (Figure 3). This either suggests that the Val-SOD2 cells are more active in LNCaP cells or there is a positive feed-back loop in Ala-SOD2 cells that is increasing superoxide. Indeed, there are reports of Val SNP being more active than Ala SNP in cryopreserved human hepatocytes [43]. Our bias is for a positive feed-back loop as NF-κB activity is increased in Ala-SOD2 cells (data not shown), and NF-κB is known to increase superoxide. Further studies are underway to confirm these findings. Interestingly, there was more SOD2 protein expression in Ala-SOD2 SNP-overexpressing cells compared to Val-SOD2. This is consistent with previous findings which similarly showed higher SOD2 expression in three breast cancer cell lines expressing Ala-SOD2 compared to three breast cancer cell lines with Val-SOD2 [42]. However, this study did not include differential mechanism(s) of action. Of note, MDA-PCa-2b is the only cell line we had that displayed the Ala/Ala genotype but did not express more SOD2 protein; we would need more cell lines with this genotype for comparisons.

We hypothesized that the increased superoxide in Ala-SOD2 SNP-overexpressed cells is responsible for the EMT, so we utilized MSKE, a natural agent that has been demonstrated to inhibit superoxide and reverse EMT [26,27]. In the present study, three-day treatment with MSKE was shown to inhibit EMT in SOD2 SNP-overexpressed cells, as indicated by decreased Snail and vimentin and increased E-cadherin (Figure 4). In our previous laboratory studies, we showed that treatment with 5 µg/mL MSKE did not affect cell viability, while treatment with 20 µg/mL (same treatment used in the present study) led to a significant decrease in cell viability [31]. Although not as effective as treatment with 5 µg/mL MSKE treatment, 20 µg/mL MSKE was also shown to revert EMT increased re-expression of E-cadherin and decreased expression in vimentin. [31] While the cell viability assays in the present study did not follow the same trend as our previous studies, which showed that MSKE alone could inhibit cell viability in C4-2 [44] and ARCaP cells overexpressing Snail [31]; partial EMT reversion was observed in this study, as treatment with MSKE alone did not completely reverse EMT indicated by reversal of EMT marker expression, but not cell migration (Figure 5). This may highlight differences observed with different cell lines. We were only able to observe decreased cell viability when a stressor, H_2_O_2,_ was used alone or combined with MSKE. Paradoxically, Ala-SOD2 SNP cells did not experience a decrease in migration following treatment with MSKE alone; instead, migration increased; this warrants further investigation. The effect of combination treatment with H_2_O_2_ and MSKE was particularly interesting. Many chemotherapeutic drugs, like Cyclophosphamide, for example, are known to cause oxidative stress within the body by generating H_2_O_2_ [37]. Using H_2_O_2_ as a representation for chemotherapy showed that chemotherapeutic drugs may be more useful in combination with exogenous antioxidants, like MSKE, given subsequent to chemotherapy.

The presence of SOD2 polymorphisms is clearly implicated in several diseases; however, evidence on differential mechanism(s) of action of SOD2 SNPs is still elusive. Consistent with our findings, many studies reported that the Ala-SOD2 leads to increased risk of prostate [21,45] and breast cancer [24,46,47], and we suggest that one mechanism may be induction of higher EMT by Ala-SOD2. In addition, a breast cancer study demonstrated that there was a positive correlation between SOD2 and EMT in breast cancer cells. Specifically, SOD2 knockdown led to EMT reversal [46]. In many studies, individuals with the *Ala/Ala* genotype were subject to either higher risk of prostate or breast cancer [21,22,23,24]. For example, a small breast cancer study studied the Val16Ala polymorphism in Shanghai Breast Cancer cases and determined that breast cancer risk was slightly elevated in premenopausal women with the *Ala/Ala* genotype, as compared with women with the *Val/Val* genotype [24]. Another study of PCa examined the relationship of SOD2 SNPs and antioxidant level status. In general, there was little overall association between SOD2 SNPs and PCa risk [21] However, men with the *Ala/Ala* genotype with high selenium levels were associated with aggressive PCa [21]. Paradoxically, data from the Mikhak, et al. study suggested that the there was no association between SOD2 polymorphisms (*Ala/Ala* and *Val/Val*) and overall PCa risk [14]. However, they did report that men with the *Ala/Ala* genotype who had low lycopene status had a two-fold increased risk of PCa compared to men with the *Val/Val* genotype [14]. These studies suggest a complex interaction between endogenous SOD2, that has become a prooxidant, and exogenous antioxidants, such as lycopene and MSKE, that was utilized in this study. Finally, meta-analysis from PubMed and EMBASE databases and relevant cancer studies determined that there was no relationship or association of risk between SOD2 and breast cancer [48]. We observed that the outlined studies were all conducted clinically in patients; and the in vivo and in vitro differences may play a critical role. Experimentation with SOD2 may translate differently with the in vitro experimentation. Concentrations for treatment with antioxidants differ from study to study, and there has been no consistent conclusion on the effect of SOD2 on cancer. In the future we plan to conduct more experimentation using organoids. The use of these 3D organoids may provide more insight to what is seen in vivo. Overall, in this study, we determined that our LNCaP SOD2 Ala-SOD2 SNP model cells were associated with higher superoxide and more induction of EMT, indicated by higher levels of Snail and vimentin, lower E-cadherin, and increased cell migration, compared to those overexpressing Val-SOD2 or Neo control. The Ala-SOD2 SNP cells were shown to be more sensitive to co-treatment with H_2_O_2_ and MSKE leading to reduced cell growth. Treatment with MSKE alone was shown to inhibit SOD2 SNP-mediated EMT marker expression in Ala-SOD2 cells, but more significantly in Val-SOD2 cells. However, surprisingly, it did not decrease cell migration. Moreover, unlike previous research, in our study 20 µg/mL MSKE alone did not promote apoptosis in either SOD2-SNP (*Ala/Ala* or *Val/Val*), but did so in the presence of H_2_O_2_ alone and in combination with MSKE. Therefore, we believe that combination H_2_O_2_-generating chemotherapeutic treatment of PCa with antioxidants like MSKE, that targets superoxide but not H_2_O_2_, may be a possible therapeutic target for PCa patients with the *Ala/Ala* genotype of SOD2.

## Figures and Tables

**Table 1:** Overview of SOD2 single-nucleotide polymorphisms (SNPs) in prostate cancer cell lines. Genomic DNA from PCa cell lines were analyzed for SOD2 SNPs with several cell lines having extra copies of alanine (Ala) and valine (Val) alleles.

Cell Line	Origin	AR	Tumorigenic Potential	SOD2 Pyrosequencing Results	Genotype
RWPE-1	Caucasian	+	None	Heterozygous C/T	Ala/Val
LNCaP	Caucasian	+	Low	Heterozygous C/T	Val/Val/Ala
PC-3	Caucasian	−	High	Heterozygous C/T	Ala/Val
ARCaP E	Caucasian	+	High	Heterozygous C/T	Ala/Ala/Val
ARCaP M	Caucasian	+	Very high	Heterozygous C/T	Ala/Val/Val
C4-2	Caucasian	+	High	Heterozygous C/T	Val/Val/Ala
MDA-PCa-2a	African American	+	High	Homozygous WT C/C	Ala/Ala
MDA-PCa-2b	African American	+	High	Homozygous WT C/C	Ala/Ala

## Data Availability

Data is contained within this article or supplementary material.

## Supplementary Materials

The following are available online at https://www.mdpi.com/2076-3921/10/2/213/s1.
