# Supplementary material for: Val16A SOD2 Polymorphism Promotes Epithelial–Mesenchymal Transition Antagonized by Muscadine Grape Skin Extract in Prostate Cancer Cells
Source: Antioxidants (Basel). 2021 Feb 1;10(2):213. doi: 10.3390/antiox10020213 (PMC7912849; doi:10.3390/antiox10020213)
Supplement: Supplementary file 1 [file antioxidants-10-00213-s001.pdf]

antioxidants

Supplementary Material

Val16A SOD2 Polymorphism Promotes Epithelial Mesenchymal Transition Antagonized by Muscadine Grape Skin Extract in Prostate Cancer Cells

Janae D. Sweeney[1], Marija Debeljak[2], Stacy Riel[2], Ana Cecilia Millena[1], James R. Eshleman[2], Channing J. Paller[3] and Valerie Odero-Marah[1,*]

[1]Center for Cancer Research and Therapeutic Development and Department of Biological Sciences, Clark Atlanta University, Atlanta, GA 30314, USA; janae.sweeney@students.cau.edu (J.D.S.); amillena@cau.edu (A.C.M.)
[2]Department of Pathology, Department of Oncology, The Sol Goldman Pancreatic Cancer Research Center, Johns Hopkins University School of Medicine, Baltimore, MD 21287, USA; mdebelj1@jhmi.edu (M.D.); Stacy_Riel@biocerna.com (S.R.); jeshlem@jhmi.edu (J.R.E.)
[3]The Sidney Kimmel Comprehensive Cancer Center, Johns Hopkins University School of Medicine, Baltimore, MD 21231, USA; cpaller1@jhmi.edu
*Correspondence: vodero-marah@cau.edu

1. Materials and Methods

1.1 SOD2 SNP Transient Overexpression

LNCaP cells were transiently transfected using Turbofect Transfection Reagent (Thermo Fisher Scientific, Waltham, MA) according to manufacturer's instructions. Briefly, 5 x 10^5 LNCaP cells were plated in 6-well plates overnight. Subsequently, cells were transfected for 48-72 hours with 4 µg Neo (empty vector), Ala-SOD2, or Val-SOD2 cDNA. Success of transfections were confirmed through probing for the DKYDDDDK FLAG-tag antibody (Genscript, Piscataway, NJ) by western blot analysis.

1.2 Western Blot Analysis

Whole protein cell lysates were collected in Lysis buffer (1X modified RIPA buffer, 1X Protease inhibitor, 1mM PMSF, and 1mM sodium orthovanadate). For each protein sample, 30 µg protein was run on a 10% SDS-PAGE gel and transferred to 0.45 µm nitrocellulose membrane (Bio-Rad, Hercules, CA). The nitrocellulose membranes were then blocked in 5% Milk (Quality Biological, Inc, Gaithersburg, MD) with TBS (1M Tris, 5M NaCl, 0.05% BSA) or 5% Milk with TBST (TBS + 0.5% Tween-20); then subsequently incubated with appropriate primary antibodies overnight in 4°C. Membranes were incubated at room temperature for 2 hours with the appropriate secondary antibody followed by visualization using Luminata Forte Western HRP Substrate (Millipore-Sigma, Burlington, MA) and visualized by Bio-Rad ChemiDoc Imaging System to detect protein expression. The membranes were stripped using mild stripping buffer (0.75% Glycine, 0.05% SDS, 0.5% Tween-20 at pH 2.2) prior to re-probing with a different antibody.

1.3 Immunofluorescence

LNCaP cells overexpressing SOD2 SNPs (5 x 10^3) were plated in 8-well chamber slide (Thermo Fisher Scientific, Waltham, MA) overnight, transiently transfected with Ala- or Val-SOD2 cDNA for 48 hours, before being fixed with 1:1 methanol/ethanol solution. Following fixation, cells were washed with 1X PBS and blocked with 5% goat serum. Subsequently, slides were incubated for 1 hour with primary antibody SOD2 in antibody diluent solution (Dako, Carpinteria, CA). Slides were then incubated with secondary antibody anti-rabbit (Oregon Green 488) for 1 hour in the dark at room temperature, before being incubated with MitoSOX to visualize the production of superoxide

in the mitochondria for 10 minutes. Slides were then counterstained with DAPI (1µg/ml; Santa Cruz Biotechnology, Dallas, TX). Slides were mounted using Fluoro-Gel mounting medium (Electron Microscopy Sciences, Hatfield, PA) and fluorescence microscopy performed using Zeiss microscope and ZEN Imaging Software. Quantitative measurement of fluorescence from was performed by scanning FLAG-SOD2 and MitoSOX fluorescent-stained cells at 40X objective on a Carl Zeiss LSM 700 laser scanning confocal microscope using Zeiss Zen 2010 Image Acquisition Software. Images were acquired from slides prepared from the same experimental conditions and same microscope settings such as digital gains, exposure and background. Mean fluorescence intensities were calculated automatically by the software. Plotted data were derived from three fields of each slide.

1.4 Cell Viability Assay

Cells were seeded at a density of 2 x 10^3 cells/well in 96-well plates and allowed to attach overnight. Cell viability was assessed according to the manufacturer's protocol using the CellTiter 96® Aqueous One Solution Cell Proliferation Assay (Promega, Madison, WI). Viability was measured at Day 0, 1, and 3 using the BioTek Synergy H1 Hybrid Reader at 490 nm absorbance.

1.5 Cell Migration Assay

Cell migration was analyzed utilizing Costar 24-well plates (Corning, Corning, NY) containing polycarbonate filter inserts with an 8 µm pore diameter size (Fisher Scientific, Pittsburgh, PA). These wells were coated with 4.46 µg/µl rat tail collagen I (BD Biosciences, San Jose, CA) on the outside and incubated for 24 hours at 4°C. 5 x 10^4 were plated in the upper chamber containing phenol/serum-free RPMI 1640 containing 5% dextran charcoal stripped fetal bovine serum (DCC); whereas the bottom chamber contained phenol/serum-free RPMI 1640 supplemented with 5% dextran charcoal stripped fetal bovine serum (DCC) and various experimental conditions which included: untreated, ethanol control (EtOH; 0.005%), H_2O_2 (250 µM), MSKE (20 µg/ml), and 250 µM H_2O_2 + 20 µg/ml MSKE. After 48 hours, cells that migrated to the bottom of the insert were fixed with 10% Formalin (Formaldehyde and 1X PBS), stained with 0.05% Crystal Violet, and counted to obtain the relative cell migration.

2. Results

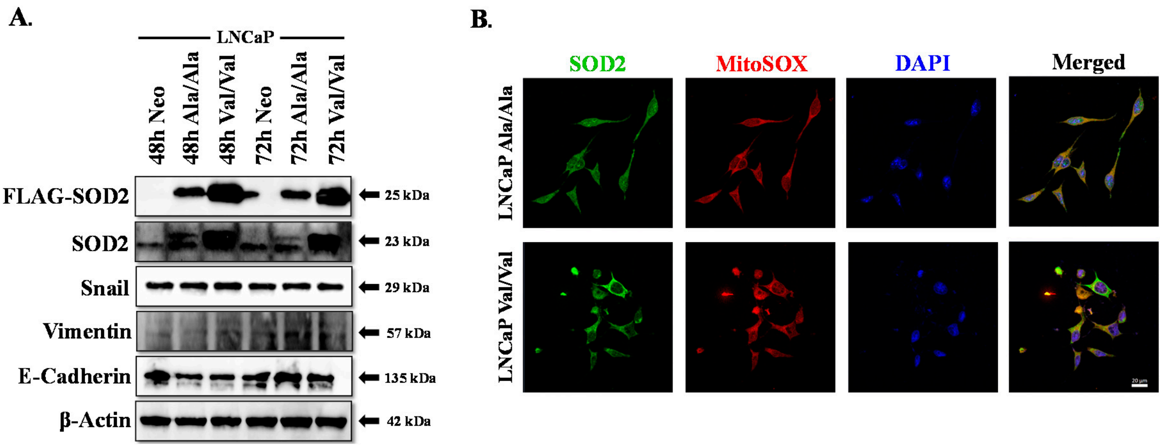

Supplementary Figure 1. Successful transient overexpression of SOD2 SNPs observed in prostate cancer cells. Ala-SOD2 (*Ala/Ala*) and Val-SOD2 (*Val/Val*) cDNA, and empty vector (Neo), were transiently transfected into LNCaP cells. **(A)** Western blot analysis of whole cell lysate showed successful overexpression of exogenous SOD2 as shown by utilizing FLAG antibody (FLAG-SOD2). **(B)** Immunofluorescence was performed with fluorescent dyes MitoSOX (red; superoxide),

Oregon Green (green; SOD2 antibody), and DAPI (blue; nuclear expression). Magnification 63X. Scale Bar= 20μm

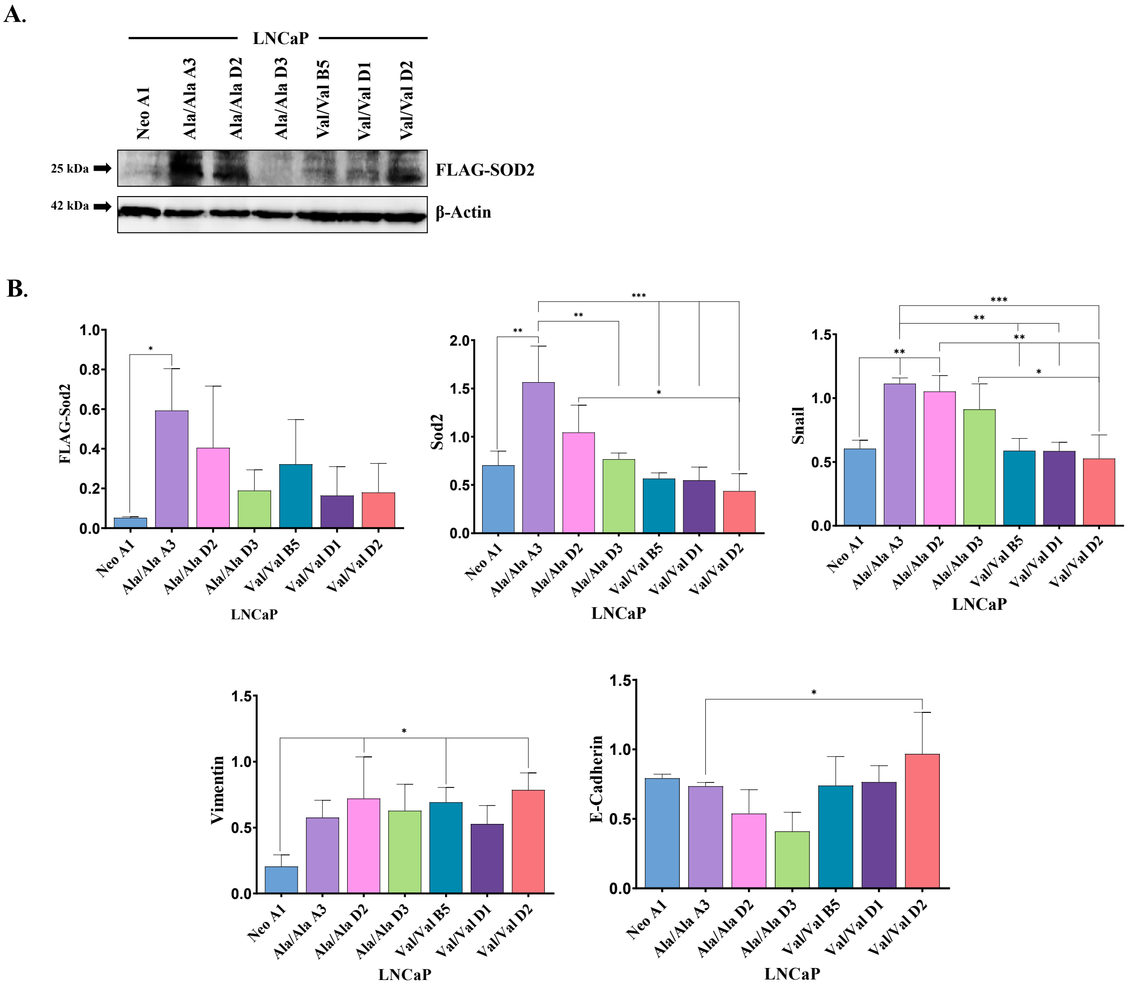

Supplementary Figure 2. Overexpression of Ala-SOD2 SNP in LNCaP cells leads to increased EMT. **(A)** Western blot analysis of whole cell lysate using FLAG-SOD2 rerun for a longer exposure of western blot was performed to demonstrate expression (supplement of Figure 2A). **(B)** Quantification of western blot in manuscript Figure 2A was performed using Image J (NIH) normalized to β-actin. Data was analyzed using GraphPad Prism, $p < 0.05$ (*), $p < 0.01$ (**), $p < 0.001$ (***).

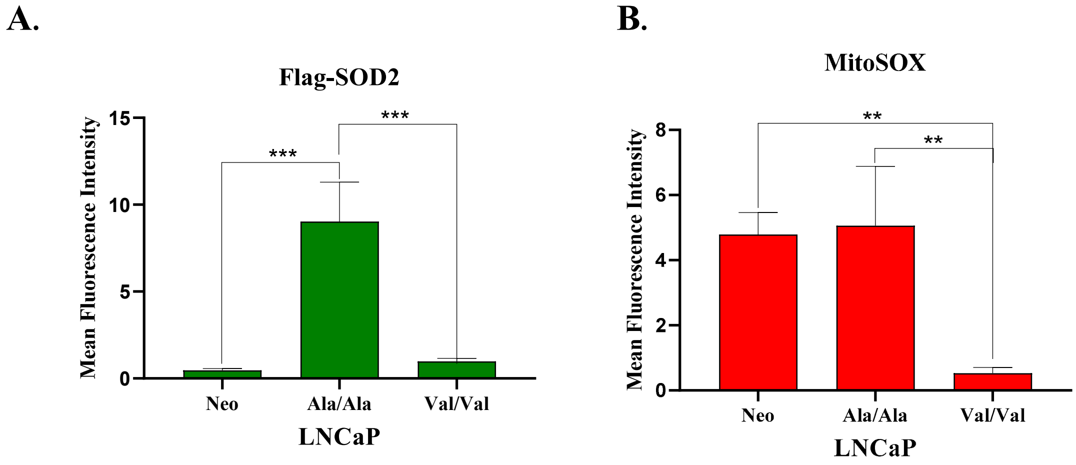

Supplementary Figure 3. SOD2 levels in LNCaP cells with stable overexpression of Ala-SOD2 SNP compared to Val-SOD2 SNP. Mean Fluorescence Intensity and Fold Change of LNCaP Neo and SOD2 SNP-overexpressing cells which were utilized for immunofluorescent analysis of **(A)** exogenous SOD2 using FLAG antibody (green) and **(B)** superoxide using MitoSOX stain (red). Data was analyzed using GraphPad Prism, $p < 0.01$ (**), $p < 0.001$ (***).

Supplementary Table 1. Image J quantification of treatment in LNCaP cells overexpressed with SOD2 SNPs.

Antibody	Day 0 Sample	Image J Quantification
Snail	LNCaP Neo EtOH	1.186
	LNCaP Neo H_2O_2	1.313
	LNCaP Neo MSKE	1.220
	LNCaP Neo H_2O_2 + MSKE	1.447
	LNCaP Ala/Ala EtOH	0.831
	LNCaP Ala/Ala H_2O_2	0.876
	LNCaP Ala/Ala MSKE	0.892
	LNCaP Ala/Ala H_2O_2 + MSKE	0.879
	LNCaP Val/Val EtOH	0.715
	LNCaP Val/Val H_2O_2	0.993
	LNCaP Val/Val MSKE	0.948
	LNCaP Val/Val H_2O_2 + MSKE	1.115
Vimentin	LNCaP Neo EtOH	0.272
	LNCaP Neo H_2O_2	0.408
	LNCaP Neo MSKE	0.251
	LNCaP Neo H_2O_2 + MSKE	0.404
	LNCaP Ala/Ala EtOH	0.342
	LNCaP Ala/Ala H_2O_2	0.626
	LNCaP Ala/Ala MSKE	0.314
	LNCaP Ala/Ala H_2O_2 + MSKE	0.444
	LNCaP Val/Val EtOH	0.364
	LNCaP Val/Val H_2O_2	0.371
	LNCaP Val/Val MSKE	0.317
	LNCaP Val/Val H_2O_2 + MSKE	0.317
E-Cadherin	LNCaP Neo EtOH	0.941

	LNCaP Neo H_2O_2	0.897
	LNCaP Neo MSKE	0.987
	LNCaP Neo H_2O_2 + MSKE	1.1017
	LNCaP Ala/Ala EtOH	0.681
	LNCaP Ala/Ala H_2O_2	0.785
	LNCaP Ala/Ala MSKE	0.960
	LNCaP Ala/Ala H_2O_2 + MSKE	1.275
	LNCaP Val/Val EtOH	0.764
	LNCaP Val/Val H_2O_2	0.778
	LNCaP Val/Val MSKE	0.876
	LNCaP Val/Val H_2O_2 + MSKE	0.828

**Antibody**	**Day 3 Sample**	**Image J Quantification**
**Snail**	LNCaP Neo EtOH	0.494
	LNCaP Neo H_2O_2	1.116
	LNCaP Neo MSKE	0.646
	LNCaP Neo H_2O_2 + MSKE	1.652
	LNCaP Ala/Ala EtOH	0.636
	LNCaP Ala/Ala H_2O_2	1.527
	LNCaP Ala/Ala MSKE	0.484
	LNCaP Ala/Ala H_2O_2 + MSKE	1.529
	LNCaP Val/Val EtOH	0.276
	LNCaP Val/Val H_2O_2	1.179
	LNCaP Val/Val MSKE	0.239
	LNCaP Val/Val H_2O_2 + MSKE	0.956
**Vimentin**	LNCaP Neo EtOH	0.208
	LNCaP Neo H_2O_2	0.420
	LNCaP Neo MSKE	0.129
	LNCaP Neo H_2O_2 + MSKE	0.464
	LNCaP Ala/Ala EtOH	0.328
	LNCaP Ala/Ala H_2O_2	0.486
	LNCaP Ala/Ala MSKE	0.260
	LNCaP Ala/Ala H_2O_2 + MSKE	0.470
	LNCaP Val/Val EtOH	1.089
	LNCaP Val/Val H_2O_2	0.814
	LNCaP Val/Val MSKE	0.774
	LNCaP Val/Val H_2O_2 + MSKE	1.335
**E-Cadherin**	LNCaP Neo EtOH	0.980
	LNCaP Neo H_2O_2	0.849
	LNCaP Neo MSKE	1.207
	LNCaP Neo H_2O_2 + MSKE	0.962
	LNCaP Ala/Ala EtOH	0.975
	LNCaP Ala/Ala H_2O_2	1.186
	LNCaP Ala/Ala MSKE	1.192
	LNCaP Ala/Ala H_2O_2 + MSKE	1.233
	LNCaP Val/Val EtOH	1.439
	LNCaP Val/Val H_2O_2	1.324
	LNCaP Val/Val MSKE	1.267
	LNCaP Val/Val H_2O_2 + MSKE	1.469

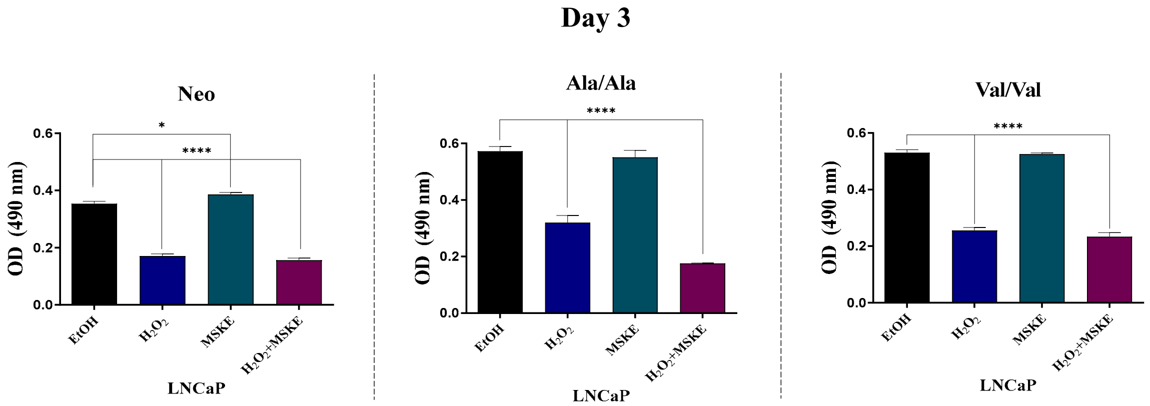

Supplementary Figure 4. Cell viability is affected by H_2O_2 and MSKE in LNCaP cells overexpressing SOD2 SNPs. MTS cell viability in LNCaP cells overexpressing Ala- (*Ala/Ala*) or Val-SOD2 (*Val/Val*) was measured following treatment with H_2O_2 and/or MSKE; treatment with H_2O_2 and MSKE alone and in combination were shown to significantly decrease cell viability. Data was analyzed using GraphPad Prism, $p < 0.05$ (*), $p < 0.0001$ (****). Figure is a representative of experiment done in triplicate at least twice independently.

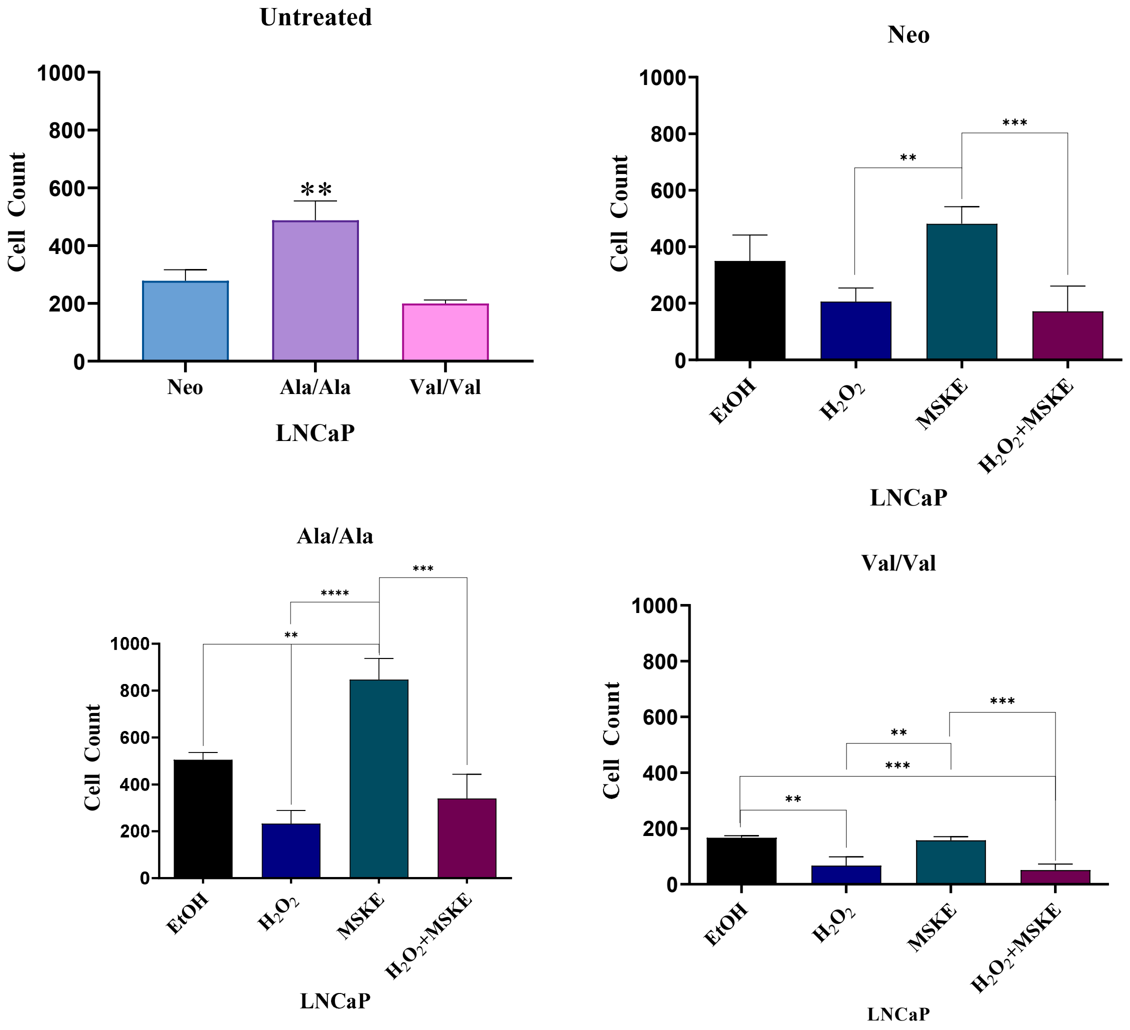

Supplementary Figure 5. Rate of cell migration is affected by combination treatment of H_2O_2 and MSKE in LNCaP SOD2 SNP-overexpressing cells. Cell migration was determined in LNCaP cells overexpressed with SOD2-SNP at baseline and following treatments; across transwells coated with type I rat-tail collagen. Data was analyzed using GraphPad Prism, $p < 0.01$ (**), $p < 0.001$ (***), $p < 0.0001$ (****). Results are representative of experiments performed in triplicate at least twice independently.
